# Diagnostic accuracy of serum protein induced by vitamin K absence (PIVKA‐II), serum a‐fetoprotein and their combination for hepatocellular carcinoma among Caucasian cirrhotic patients with diagnostic or non‐diagnostic serum a‐fetoprotein levels

**DOI:** 10.1002/cam4.6825

**Published:** 2024-02-15

**Authors:** Antonia Syriha, Spyridon Pantzios, Dionysia Mandilara, Petros Galanis, Ioanna Stathopoulou, Georgia Barla, Ioannis Elefsiniotis

**Affiliations:** ^1^ Academic Department of Internal Medicine—Hepatogastroenterology Unit, General and Oncology Hospital of Kifisia “Agioi Anargyroi” National and Kapodistrian University of Athens Athens Greece

**Keywords:** AFP, biomarkers, cirrhosis, HCC, PIVKA‐II

## Abstract

**Aim:**

The aim of our study was to evaluate the accuracy of serum biomarkers (AFP/PIVKA‐II) and their combination in HCC diagnosis among Caucasian cirrhotic patients.

**Methods:**

Serum AFP/PIVKA‐II levels were evaluated in 218 cirrhotics (163 males, 118 CTP‐A, 66 ALBI‐I, 111 with varices, 63 with diabetes) with (*n* = 90) or without (*n* = 128) HCC. Patients with HCC were categorized to BCLC Stage 0/A (*n* = 12), B (*n* = 21), C (*n* = 48), and D (*n* = 9).

**Results:**

The two groups were comparable for all baseline parameters except for age, platelets, and diabetes presence. Median levels of AFP (239.1 vs. 4.0 ng/mL) and PIVKA‐II (4082.7 vs. 45.8 mAU/mL) were both significantly higher in HCC group compared to controls (*p* < 0.001). AUROC and cutoff value for HCC diagnosis were 88%/12.35 ng/mL (AFP) and 84.4%/677.13 mAU/mL (PIVKA‐II), whereas their combination showed better diagnostic accuracy (AUROC = 90.2%). The diagnostic accuracy of each biomarker separately was moderate or good in BCLC‐0/A/B and was excellent only for BCLC‐C patients (AFP: AUROC = 94.3%, cutoff = 12.35 ng/mL and PIVKA‐II: 91.3%, 253.51 mAU/mL) whereas their combination presented quite acceptable results in BCLC‐B (AUROC = 92.4%) and BCLC‐C (AUROC = 95.7%). Excluding HCC patients with high AFP (above 400 ng/mL), the diagnostic accuracy of each biomarker separately and their combination was moderate/good in all groups, except for their combination in BCLC‐C (AUROC = 90.5%).

**Conclusions:**

Each biomarker separately showed acceptable accuracy for detecting HCC in cirrhotic patients and excellent for those in BCLC‐C stage. The combination of the biomarkers presented excellent results in BCLC‐B/C patients. The diagnostic accuracy of PIVKA‐II and the combination of the two biomarkers in patients expressing low/non‐diagnostic AFP levels was good in BCLC‐B and excellent in BCLC‐C patients.

## 1 INTRODUCTION

Hepatocellular carcinoma (HCC) is the most frequent cancer that affects the liver, accounting for approximately 75% of all cases.^1^ Approximately one‐fifth of patients with chronic liver disease will be diagnosed with liver cancer once during their lifetime. Risk of HCC development varies according to cause, geographic data, family HCC history, and liver disease severity.^2^ It is well known that cirrhosis of any underlying etiology represents an important risk factor for tumor formation.^3^

Annual HCC development rate is approximately 1%–8% in cirrhotic patients, especially in those who exhibit features of decompensated liver disease and/or significant portal hypertension and thus all cirrhotic patients should be entered into HCC surveillance programs.^3^ HCC detection in early stages provides clinicians with the opportunity to treat HCC using potentially curative interventions.^4^ Surveillance should also continue for cirrhotics in whom the etiological factor of chronic liver disease has been cured, such as viral hepatitis C.^5^, ^6^ Liver ultrasound (US) every 6 months, with or without concomitant use of serum a‐fetoprotein (AFP) levels, is strongly recommended by EASL/AASLD/APASL guidelines for all cirrhotic patients participating in HCC surveillance programs.^7^, ^8^, ^9^ US limitations in the early detection of HCC have been recognized in many studies, particularly in obese patients with nonalcoholic fatty liver disease (NAFLD).^10^, ^11^ US alone has low sensitivity in early HCC detection, which is a fact that emphasizes the importance of determining whether other serum biomarkers might complement AFP and US in the surveillance setting.^11^, ^12^ Data concerning the utility of several proposed tumor biomarkers and/or their combination for accurate early HCC detection in cirrhotic patients is still needed.

Protein‐Induced‐by‐Vitamin‐K‐Absence or‐Antagonist‐II (PIVKA II), also known as des‐gamma carboxy prothrombin (DCP), is a precursor of prothrombin and plays a key role in hepatocarcinogenesis.^13^ Studies have shown that PIVKA‐II could be a more specific HCC biomarker than AFP, albeit with lower sensitivity. Serum PIVKA‐II levels in HCC patients are significantly elevated compared to healthy controls, which suggests that PIVKA‐II could be a useful complement to AFP in HCC diagnosis.^13^, ^14^ Moreover, the addition of serum PIVKA‐II levels to routine AFP test might provide a more suitable biomarker approach to detect HCV‐induced HCC in patients with chronic HCV infection.^15^ The clinical value of the combination of the two biomarkers has already been studied in small regional studies and seems to be superior compared to each test alone for the diagnosis of HCC, according to a recent study from China.^16^ On the other hand, data from prospective Phase III studies concerning the utility of serum PIVKA‐II levels in the diagnosis of HCC among Caucasian patients with liver cirrhosis of any etiology, which is mainly the target population of HCC surveillance, are scarce^17^, ^18^ as well as information regarding the diagnostic accuracy of each biomarker separately and their combination in HCC patients according to the stage of the malignant disease. The observation that there is significant overlap between the values of both biomarkers among patients with advanced liver disease, with or without concomitant HCC, highlights the importance of the evaluation of their diagnostic accuracy in this group of patients, taking into consideration the singularities of HCC and liver cirrhosis.^19^

The aim of our study was to evaluate the diagnostic accuracy of serum AFP and PIVKA‐II or their combination in HCC diagnosis among Caucasian patients with compensated or decompensated liver cirrhosis of various etiologies.

## 2 METHODS

### 2.1 Patients

We prospectively evaluated serum AFP and PIVKA‐II levels in Caucasian patients with compensated or decompensated liver cirrhosis who consecutively presented with or without concomitant HCC in the outpatient Hepatology Unit of General and Oncology Hospital of Kifisia “Oi Agioi Anargyroi.” Inclusion of patients in the study commenced in September 2016 and ended in December 2022. All patients with liver cirrhosis who participated in the current study experienced chronic liver disease of various etiologies and different stages of liver disease, given the fact that both compensated and decompensated cirrhotics were included in the study. The presence of cirrhosis was evaluated in the vast majority of patients using transient elastography and in a small proportion of patients liver biopsy was performed to define the diagnosis of cirrhosis. Medical history and standard hematology and biochemical tests were used to assess the baseline characteristics of all patients. HCV‐related cirrhotic patients who participated in the study had received treatment with DAAs in the past and exhibited sustained virological response (undetectable HCV‐RNA 12 weeks following treatment discontinuation and at least once a year). Furthermore, all cirrhotic patients with chronic HBV infection had undetectable HBV‐DNA levels on baseline and were treated with long‐term nucleoside or nucleotide analogues (tenofovir or entecavir). Cirrhotic patients of any etiology who declared alcohol use at the time or during the last 6 months of patient screening were excluded, as well as those taking vitamin K antagonist or other anticoagulants.

Decompensated liver cirrhosis was defined as the presence of at least one liver‐related complication from patient history, such as acute variceal bleeding, ascites with SAAG >1.1 g/dL, hepatic encephalopathy or jaundice. Absence of bacterial infection was based on clinical examination, the concomitant use of chest X radiography to rule out lower respiratory tract infections and negative blood cultures, as well as urine and ascites cultures, in order to rule out bacteremia, urinary tract, and ascitic fluid infections. Spontaneous bacterial peritonitis (SBP) was excluded in all patients who exhibited ascites after diagnostic paracentesis using the threshold of 250 neutrophils/mm^3^. The severity of chronic liver disease was assessed using well‐defined and widely‐used scores for cirrhotic patients, such as Child‐Pugh‐Turcotte (CPT) score, MELD score, and ALBI grade.^20^, ^21^

All patients included in the study had contrast‐enhanced CT or MRI assessment at least within the last 6 months prior to study entry, in order to suspect or to exclude cirrhotic patients with concurrent HCC. We suspected HCC presence in the vast majority of patients combining contrast—enhaced CT/MRI findings with laboratory values of serum AFP levels using the 400 ng/mL threshold, above of which AFP seems to have more diagnostic accuracy. All patients in whom HCC was suspected due to the presence of liver lesions in imaging, subsequently underwent liver biopsy in order to histologically confirm HCC diagnosis. Patients with documented HCC were further categorized according to the BCLC HCC staging system in patients with early‐stage HCC (BCLC stage 0/A), intermediate stage (BCLC B), advanced HCC (BCLC‐C), and finally end‐stage HCC (BCLC‐D), using the most recent 2022 BCLC update.^22^

We collected written informed consent from all patients who participated in the study. The protocol of our study was in line with the Declaration of Helsinki and was evaluated and originally approved by the Ethics Committee of the School of Health Sciences, National and Kapodistrian University of Athens, Greece.

### 2.2 Sample collection

Sample collection was performed during patients' first visit in the outpatient Hepatology Unit, at the same time as demographical data recording was done and hematology and biochemical laboratory tests were obtained. Whole blood (approximately 10 mL) was collected from peripheral veins of patients and serum as well as plasma was immediately separated and stored frozen at −80°C until further analysis. The chemiluminescent microparticle immunoassay (CMIA) was used for the quantitative determination of serum AFP (ARCHITECT AFP). Serum PIVKA‐II levels were measured using a commercially available ELISA kit (ARCHITECT PIVKA‐II immunoassay) following the specific instructions of the manufacturer.

### 2.3 Statistical analysis

Categorical variables are presented with numbers and percentages. We also use mean, median, standard deviation, minimum and maximum value to present continuous variables. Mann–Whitney test, independent samples *t*‐test and chi‐squared test were used to estimate differences between the two groups (HCC group and control cirrhotic group) regarding baseline characteristics. Moreover, we used Mann–Whitney test to compare AFP and PIVKA‐II values among the two groups as these variables did not follow the normal distribution. Diagnostic accuracy for AFP and PIVKA‐II was assessed with ROC curves. In that case, we estimated the area under the ROC curve (AUROC), standard error, 95% confidence interval, and *p*‐value. C‐statistic ranges from 0 to 1 with values lower than 0.5 indicate an unacceptable model, values between 0.5 and 0.6 indicate low discriminative power, values between 0.61 and 0.8 indicate moderate diagnostic accuracy, values between 0.81 and 0.9 indicate good diagnostic accuracy, and values higher than 0.9 indicate excellent discriminative power.^23^ We used the Youden Index^24^ to find the cutoff values of AFP and PIVKA. In particular, Youden Index is equal to (sensitivity + specificity) − 1. We used a binary logistic regression model to predict the probability of HCC in order to evaluate the diagnostic performance of the combination of AFP and PIVKA‐II combination. *p*‐Values <0.05 were considered as statistically significant. Statistical analysis was performed with the IBM SPSS 21.0 (IBM Corp. Released 2012. IBM SPSS Statistics for Windows, Version 21.0. Armonk, NY: IBM Corp.).

## 3 RESULTS

Two hundred and eighteen patients with liver cirrhosis (163 males, 118 with CPT score A, 66 ALBI Grade I, 111 with varices, and 63 with diabetes) with (*n* = 90, HCC group) or without (*n* = 128, control group) concomitant histologically confirmed HCC were evaluated for serum levels of the two biomarkers, during a programmed visit in our department. The mean age of the patients was 65 ± 11.5 years and they more often presented with metabolic dysfunction associated fatty liver disease (MAFLD, 90/218, 41.3%), with the second and third causes of cirrhosis being chronic hepatitis C (51/218, 23.4%) and B (43/218, 19.7%), respectively. Compensated and decompensated cirrhotic patients were equally distributed in our study (*n* = 109 in each group) and the mean MELD score of the whole group was 12.2 ± 6.5. Patients with HCC (*n* = 90) were categorized as Stage 0/A (*n* = 12, 13.3%), Stage B (*n* = 21, 23.3%), Stage C (*n* = 48, 53.3%), and Stage D (*n* = 9, 10%), according to BCLC staging system. The main cause of liver disease in HCC cases was MAFLD (44/90, 48%) followed by chronic HBV (25/90, 27%) and chronic HCV (17/90, 18%) infection.

The two groups of cirrhotic patients were comparable for the majority of baseline characteristics evaluated such as gender (*p* = 0.07), liver decompensation (*p* = 0.4), CPT score (*p* = 0.8), ALBI grade (*p* = 0.4), presence of varices (*p* = 0.2), MELD score (*p* = 0.8), and viral or nonviral etiology of chronic liver disease (*p* = 0.4), except for age, platelet count and presence of diabetes, as shown in Table 1. HCC cirrhotic patients were older (mean age 68.5 vs. 62.5 years, *p* < 0.001), had higher platelet count (198.100 vs. 139.900, *p* < 0.001) and presented frequently with diabetes (52.4% vs. 47.6%, *p* = 0.03) compared to cirrhotic patients without HCC.

**TABLE 1:** Baseline characteristics for the entire population of the study.

	HCC				Total			*p*‐Value
	No		Yes					
	*Ν*	%	*Ν*	%	*Ν*	%		
Gender								0.07
Male	90	55.2	73	44.8	163	74.8		
Female	38	69.1	17	30.9	55	25.2		
Age	62.5	12.1	68.5	9.6	65.0	11.5		<0.001
Diabetes								0.03
No	98	63.2	57	36,8	155	71.1		
Yes	30	47.6	33	52.4	63	28.9		
Decompensation								0.4
No	61	56	48	44	109	50		
Yes	67	61.5	42	38.5	109	50		
Child‐Pugh score								0.8
A	67	56.8	51	43.2	118	54.1		
B	40	59.7	27	40.3	67	30.7		
C	21	63.6	12	36.4	33	15.1		
Varices								0.2
No	58	54.2	49	45.8	107	49.1		
Yes	70	63.1	41	36.9	111	50.9		
Albi								0.4
1	43	65.2	23	34.8	66	30.3		
2	67	54.9	55	45.1	122	56		
3	18	60	12	40	30	13.8		
Meld score	12.3	6.4	12.0	6.5	12.2	6.5		0.8
PLT	139.9	75.6	198.1	113.5	163.9	97.2		<0.001
Type								0.4
Nonviral	53	55.8	42	44.2	95	43.6		
Viral	75	34.4	48	22	123	56.4		

As expected, the median levels of AFP (239.1 vs. 4.0 ng/mL, *p* < 0.001) were significantly higher in HCC group compared to control group. The same result was also found for the median levels of PIVKA‐II (4082.7 vs. 45.8 mAU/mL, *p* < 0.001) in HCC patients compared to cirrhotic ones without HCC (Table 2).

**TABLE 2:** Median AFP (ng/mL) and PIVKA‐II (mAU/mL) values in HCC group (HCC+) and control group (HCC−).

	Median	Minimum value	Maximum value	*p*‐Value
PIVKA‐overall	99.5	11.6	30.000	<0.001
ΗCC−	45.8	11.6	30.000	
ΗCC+	4082.7	24.8	30.000	
AFP‐overall	5.9	1.0	363.691	<0.001
ΗCC−	4.0	1.0	474	
ΗCC+	239.1	1.0	363.691	

**FIGURE 1:**
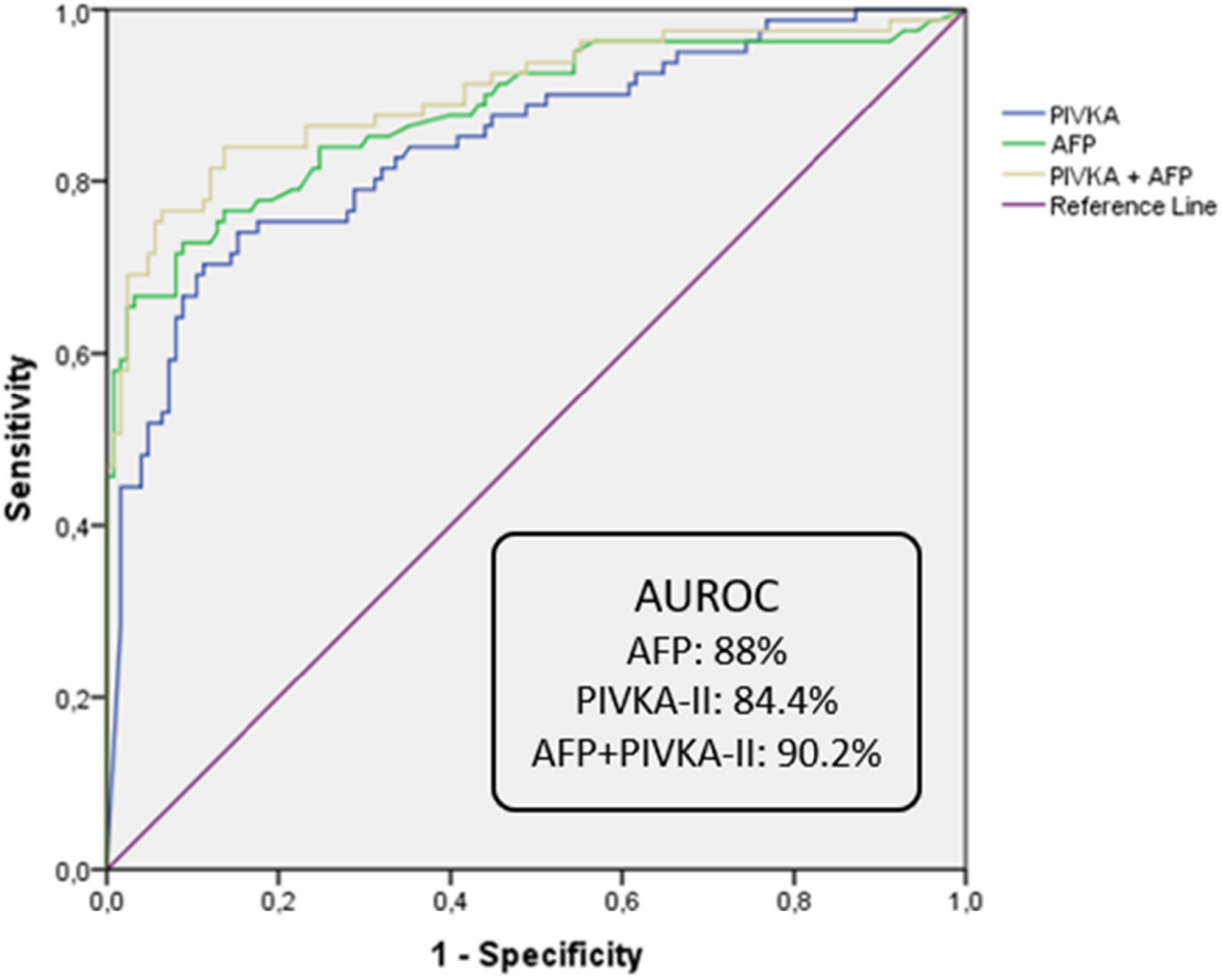
ROC curves for AFP, PIVKA‐II and their combination for the diagnosis of HCC in the whole study population.

**FIGURE 2:**
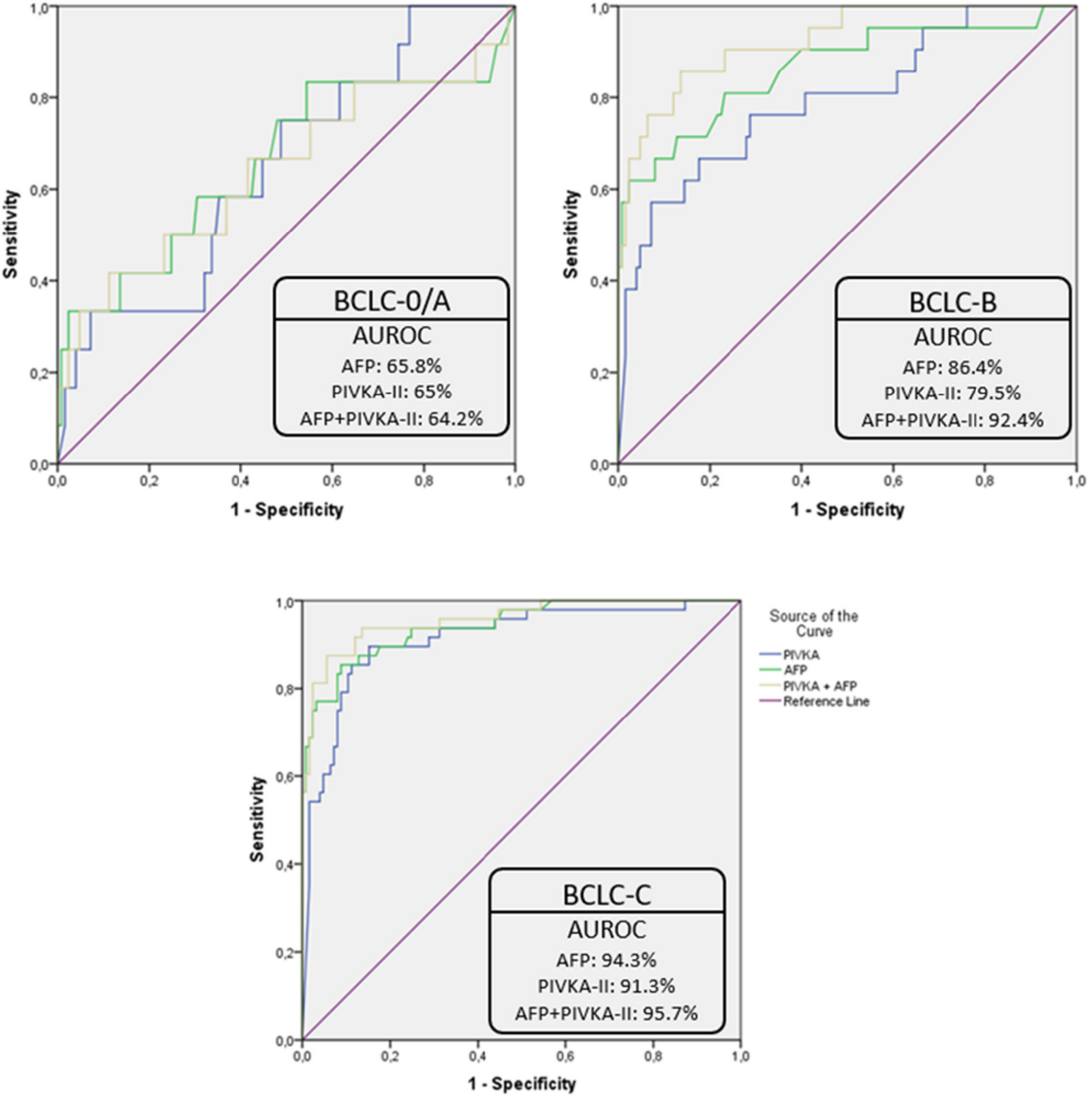
ROC curves for AFP, PIVKA‐II and their combination for the diagnosis of HCC in the study population according to BCLC stage.

The AUROC curve and the best proposed cutoff value for HCC diagnosis were 88% with 12.35 ng/mL for AFP and 84.4% with 677.13 mAU/mL for PIVKA‐II, respectively, whereas the diagnostic accuracy for HCC diagnosis was slightly better (AUROC 90.2%) with the combination of the two biomarkers (Figure 1). The diagnostic accuracy of each biomarker separately was moderate for BCLC‐0/A (AUROC 65.8% for AFP and 65% for PIVKA‐II), higher in BCLC‐B HCC patients and especially for AFP (AUROC 86.4% for AFP and 79.5% for PIVKA‐II) and quite high (AUROC above 90%) among BCLC‐C stage HCC patients (AFP: AUROC = 94.3%, best proposed cutoff value = 12.35 ng/mL and PIVKA‐II: AUROC = 91.3%, best proposed cutoff value = 253.51 mAU/mL), as shown in Figure 2. The combination of the two biomarkers presented excellent diagnostic curves in BCLC‐B (AUROC = 92.4%) as well as BCLC‐C (AUROC = 95.7%) stage HCC patients (Figure 2).

**FIGURE 3:**
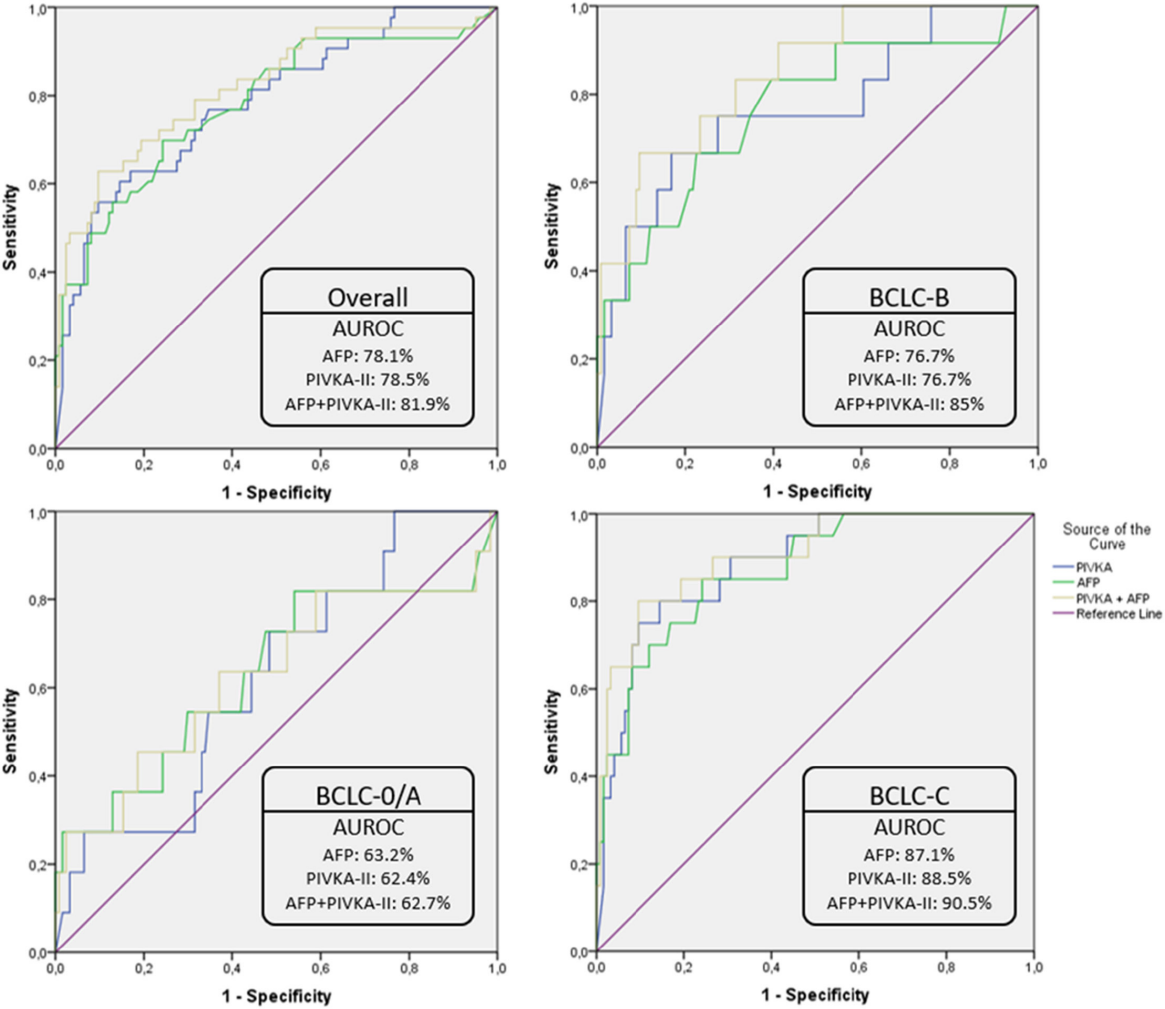
ROC curves for AFP, PIVKA‐II and their combination for the diagnosis of HCC in patients with serum AFP levels <400 ng/mL, overall and according to BCLC stage.

The significant role of elevated AFP levels (>400 ng/mL) in the diagnosis of HCC among cirrhotic patients has been clearly documented. For that reason, we tried to evaluate the diagnostic accuracy of PIVKA‐II levels in patients without diagnostic serum AFP levels. Excluding 39 HCC patients with AFP levels above 400 ng/mL, we assessed the diagnostic accuracy of each biomarker separately as well as the combination of them in HCC detection. As presented in Figure 3, in BCLC‐A patients the AUROCs for AFP, PIVKA‐II and their combination were 63.2%, 62.4%, and 62.7%, respectively. Higher diagnostic curves were seen in BCLC‐B patients (76.7% for both AFP and PIVKA‐II separately) while the combination of the two biomarkers in this category achieved an AUROC of 85%. Last but not least, in BCLC‐C patients, both biomarkers (AFP AUROC: 87.1%, PIVKA‐II AUROC: 88.5%) had very good diagnostic accuracy separately, which became excellent with their combined use (combination AUROC: 90.5%).

**FIGURE 4:**
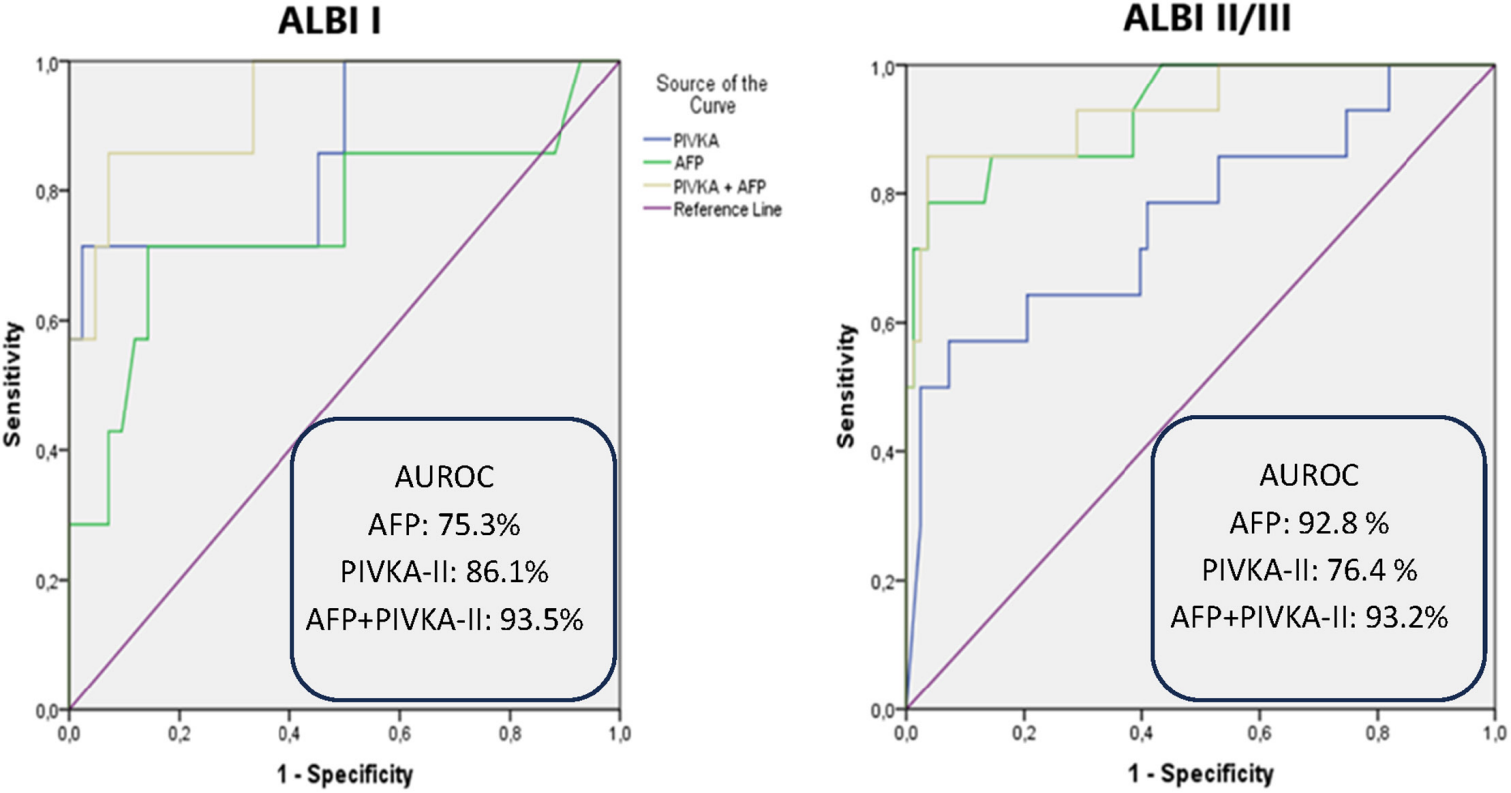
ROC curves for AFP, PIVKA‐II and their combination for the diagnosis of HCC in BCLC‐B patients according to ALBI grade.

Furthermore, taking into account that BCLC‐B is a quite heterogenic group of patients which includes patients with various tumor burden and different liver disease severity,^25^ we further assessed the diagnostic accuracy of those biomarkers in BCLC‐B patients according to ALBI classification (ALBI‐I vs. ALBI‐II/III). In our study 21 patients were categorized as BCLC‐B and if separated according to ALBI‐I (7/21) and ALBI‐II/III (14/21) we observe that in ALBI‐I BCLC‐B patients AUROC for AFP was 75.3% (best proposed cutoff value 6.75 ng/mL), for PIVKA‐II AUROC was 86.1% (best proposed cutoff value 195.19 mAU/mL) and their combination still exhibited an excellent diagnostic accuracy with an AUROC of 93.5%. However, in ALBI‐II/III patients, AFP had a quite higher diagnostic accuracy when used alone compared to ALBI‐I patients (AUROC: 92.8%, best proposed cutoff value 42.75 ng/mL), while PIVKA‐II had lower diagnostic accuracy when used alone compared to ALBI‐I patients (AUROC: 76.4%, best proposed cutoff value 3688 mAU/mL). Interestingly, when the combination of biomarkers was used in BCLC‐B patients with an ALBI grade of II or III, the diagnostic accuracy was also excellent, as in ALBI‐I BCLC‐B patients (AUROC: 93.2%) (Figure 4).

## 4 DISCUSSION

Surveillance in cirrhotic patients for the detection of HCC in early stages is of critical importance.^26^, ^27^ Abdominal US every 6 months with or without the use of serum AFP levels is the recommended practice for HCC surveillance in cirrhotic patients worldwide,^3^, ^4^, ^26^, ^27^ with a sensitivity ranging between 69% and 88% for early‐stage HCC.^26^ Despite that, inadequate US quality has been observed in advanced cirrhotic patients, especially those with high body mass index and nonalcoholic steatohepatitis as the main etiology of cirrhosis.^28^ Moreover, AFP has also relatively low sensitivity and specificity for HCC detection in early stages as well as false positive results in the setting of active necroinflammation in cirrhotic patients.^26^, ^27^ In our study we tried to evaluate the diagnostic accuracy of the biomarkers AFP and PIVKA‐II, separately or in combination, for the diagnosis of HCC among cirrhotic individuals of Caucasian origin. Our cohort included Caucasian patients with advanced chronic liver disease as approximately half of them presented with decompensated cirrhosis, Child‐Pugh stage B/C, and clinically significant portal hypertension (as suggested by the endoscopically presence of esophageal varices) whereas most of them (69.8%) belong to ALBI grade II or III. Additionally, MAFLD was observed as the main etiology of liver cirrhosis in the whole group of cirrhotic patients as well as in those with concomitant HCC. It is important to mention that as our department is considered a referral HCC center of the Athens metropolitan area as well as Southern Greece, a great percentage of our study population (90/218, 41.3%) were cirrhotic patients with HCC which does not reflect the estimated annual incidence of HCC (1%–6% per year) in cirrhotics.^28^ We prospectively collected demographic, clinical, laboratory, imaging, and histological data as well as serum samples for consecutively presented cirrhotic patients, whether they suffered from concomitant HCC or not, and subsequently assessed for the diagnostic accuracy of the two evaluated biomarkers.

Even though the median serum levels of both biomarkers were significantly higher in cirrhotic patients with HCC compared to those without HCC, the diagnostic accuracy of each biomarker for the diagnosis of HCC was estimated moderate in early‐stage HCC (AUROC 65.8% for AFP and 65% for PIVKA‐II in BCLC‐0/A patients), good in BCLC‐B stage (86.4% for AFP and 79.5% for PIVKA‐II), and was excellent among BCLC‐C stage HCC patients (AUROC 94.3% for AFP and 91.3% for PIVKA‐II). The combination presented excellent diagnostic curves only in intermediate BCLC‐B (AUROC 92.4%) and advanced BCLC‐C (AUROC 95.7%) stage HCC patients. It seems that the diagnostic accuracy of each biomarker separately for the diagnosis of HCC is not quite useful for the early stage, more useful for the intermediate stage of the disease and very useful for the advance stage in which the therapeutic options with a curative intent are more limited. Moreover, the combination of biomarkers seems to improve the diagnostic accuracy for HCC detection in the intermediate (BCLC‐B) and advanced (BCLC‐C) stage, but not in very early/early stages (BCLC‐0/A), a finding that could possibly suggest the limited role of the two evaluated biomarkers alone or in combination in the prediction of HCC among cirrhotic patients and their usefulness in surveillance programs without concomitant imaging. PIVKA‐II revealed the best predictive performance compared to AFP and AFP‐L3, in studies which evaluated HCC patients and patients with chronic hepatitis C^15^ or patients with benign liver lesions, liver metastases, and other gastrointestinal malignancies.^16^ It is important to note, that in these studies the control group was either non‐cirrhotic patients^16^ or mainly patients with compensated Child‐Pugh A cirrhosis as presented in the study by Liu S et al.,^15^ in contrast to our study in which approximately half of the study population exhibited advanced decompensated liver cirrhosis. Non‐cirrhotic control groups may overestimate the diagnostic accuracy of each individual biomarker as well as the combination of them in the detection of HCC, as it has been observed that liver cirrhosis significantly impacts serum PIVKA‐II and AFP levels.^13^, ^14^

Patients with HCC who are classified in the BCLC‐B stage are quite different in many aspects (tumor burden, liver disease severity, comorbidities etc.), a fact that suggests the great heterogeneity of this HCC group.^25^ BCLC‐B patients are characterized by different HCC burden (from huge single nodules to multinodular with diameters above the widely accepted according to Milan criteria) and different liver disease severity according to ALBI grade and so it seems quite reasonable to assess the diagnostic accuracy of these biomarkers in BCLC‐B patients according to ALBI grade. We found that the diagnostic accuracy of the combination of the two biomarkers remained excellent in BCLC‐B patients, irrespective of their ALBI status, a finding that should be re‐evaluated, and validated in larger BCLC‐B cohorts.

Studies that evaluated only patients with liver cirrhosis concluded that PIVKA‐II is a useful biomarker for diagnostic characterization of liver nodules and it provides higher diagnostic accuracy for HCC when combined with AFP.^17^, ^18^ It is of great importance that in the study by Saitta et al.,^17^ which evaluated only cirrhotic patients, the sensitivity, specificity and AUROC of PIVKA‐II was 60%,88%, and 71%, respectively and increased to 70%, 94%, and 76.4% when combined with AFP. These results are in accordance with our results which conclude that the diagnostic accuracy of each biomarker individually for HCC detection is suboptimal (<90%) when patients with advanced cirrhosis and severe liver function impairment or portal hypertension are evaluated, whereas the combination of the two biomarkers produces quite better results especially in the HCC diagnosis of patients with intermediate or advanced stages. Studies suggest that AFP and PIVKA‐II are complementary biomarkers as their production occurs through different pathways.^15^, ^16^, ^17^, ^18^ In patients with very early/early HCC the diagnostic accuracy of each biomarker was almost equal (AUROC 65.8% for AFP and 65% for PIVKA‐II) and did not improve with the combination of them (AUROC 64.2%) whereas there was an additive, complementary effect of PIVKA‐II to AFP in the HCC diagnosis in intermediate and advanced stages, according to the results of our study. It seems that the combination of the two biomarkers is not the most useful tool available to predict the presence of very early or early HCC in advanced cirrhotic patients, irrespective of the imaging results. The most benefited group from the combination of the two biomarkers was intermediate (BCLC‐B) stage HCC cirrhotic patients, as in this group the diagnostic accuracy of each biomarker separately was moderate or good (AUROC 86.4% for AFP and 79.5% for PIVKA‐II) and became excellent (AUROC 92.4%) when the combination was used. Despite the additive effect observed with the combination of PIVKA‐II to AFP in BCLC‐C HCC patients (AUROC 95.7%), this could not be of major importance as each biomarker separately produces quite high results in the HCC diagnosis of advance stage patients (AUROC 94.3% for AFP and 91.3% for PIVKA‐II).

Advances in molecular classification of HCC classified it in two major subclasses: a proliferation class that includes clinically aggressive tumors which are poorly differentiated and frequently present with high AFP levels and a nonproliferation class that includes less aggressive, chromosomally stable tumors that usually do not express AFP.^26^, ^29^ In our study 39 out of 90 HCC cases (43.3%) exhibited diagnostic AFP levels (above 400 ng/mL), 24 of viral, and 15 of nonviral etiology of liver disease. According to a small pilot study from Basile et al.,^30^ AFP displayed a better diagnostic performance than PIVKA‐II for viral HCC while PIVKA‐II is better for metabolic HCC. Moreover, PIVKA‐II could be considered a strong predictor of advanced HCC with macrovascular invasion in patients with MAFLD^30^ and/or large tumors.^18^ In our study, the diagnostic performance of PIVKA‐II in patients who did not express high serum AFP levels was moderate in the whole group (AUROC 78.5%) as well as among BCLC‐0/A (AUROC 62.4%) and BCLC‐B (AUROC 76.7%) patients and was very good among HCC patients of advanced BCLC‐C stage (AUROC 88.5%). We observed that the combination of both biomarkers resulted in excellent diagnostic accuracy only among BCLC‐C patients (AUROC 90.5%), a finding which suggests that serum PIVKA‐II levels might be less useful for HCC diagnosis in early stages in patients with cirrhotic background who do not express high serum AFP levels. These findings should be re‐evaluated in studies with large numbers of cirrhotic patients with HCC with available data on molecular classification of tumors.

Our study has some limitations too. The relatively small sample size of HCC patients—especially in the early stages (only 12 patients were BCLC‐0/A)—and the absence of tumor molecular classification data are among the most mentionable. The combination of such data with histology biomarkers associated with molecular tumor profile as well as the reproduction of such methods in large worldwide multi‐center prospective cohorts could lead to some interesting results regarding the usage of such biomarkers especially for early HCC diagnosis (stages BCLC‐0/A), where curative methods such as liver resection can possibly lead to complete HCC treatment. On the other hand, the performance of such study in a single center with the same Hepatologists, Gastroenterologists, Liver Pathologists, and Liver imaging experts as well as the use of the same laboratory for all measurements, could possibly reduce biases, and somehow outweigh the aforementioned limitations.

## 5 CONCLUSION

According to our knowledge, this is the first study to assess the diagnostic accuracy of AFP, PIVKA‐II or their combination in Caucasian cirrhotic patients with or without HCC according to initial BCLC stage. As for the clinical significance of our findings, the additive value of PIVKA‐II to AFP in HCC diagnosis among cirrhotic patients was mainly observed in BCLC‐B HCC patients of the whole study population, as well as in BCLC‐B and C patients when HCC patients with very high/diagnostic AFP levels (>400 ng/mL) were excluded. These results need further validation in larger prospective cohorts.

## AUTHOR CONTRIBUTIONS

**Antonia Syriha:** Conceptualization (lead); data curation (lead); formal analysis (lead); investigation (lead); methodology (lead); project administration (lead); resources (equal); validation (equal); writing – original draft (equal). **Spyridon Pantzios:** Data curation (lead); formal analysis (equal); investigation (lead); methodology (lead); resources (equal); visualization (lead); writing – original draft (supporting); writing – review and editing (equal). **Dionysia Mandilara:** Data curation (equal); formal analysis (equal); investigation (equal); methodology (equal); project administration (equal); resources (equal). **Petros Galanis:** Data curation (equal); resources (equal); software (lead); visualization (equal); writing – original draft (equal). **Ioanna Stathopoulou:** Data curation (equal); investigation (equal); methodology (equal); validation (equal). **Georgia Barla:** Data curation (equal); investigation (equal); methodology (equal); validation (lead). **Ioannis Elefsiniotis:** Conceptualization (lead); project administration (lead); supervision (lead); writing – original draft (lead); writing – review and editing (equal).

## FUNDING INFORMATION

The study was partially supported by the Institute for the Study of Gastrointestinal Neoplasms.

## CONFLICT OF INTEREST STATEMENT

There is no conflict of interest from all authors.

## ETHICS STATEMENT

Ethics Committee of the School of Health Sciences of the National and Kapodistrian University of Athens, Greece.

## INFORMED CONSENT

Written informed consent was collected from all patients participating in the current study.

## REGISTRY AND THE REGISTRATION NO. OF THE STUDY/TRIAL

Protocol number: 399—3/6/2022.

## Data Availability

Data sharing is not applicable to this article as no new data were created or analyzed in this study.
